# To the Editors of the Medical and Physical Journal

**Published:** 1805-12

**Authors:** 


### *To the Editors of the Medical and Physical Journal.*

Gentlemen,

A Person, about the age of forty, desired my advice concerning a diseased testicle. It was nearly the size of a small pomegranate, very hard, quite free from pain; and the spermatic process free from all appearance of disease. Castration, he said, he was resolved not to yield to; and only wished to know whether I could think of any application likely to rid him of his disease. I gave him my opinion freely, on the great improbability of his being relieved by any other means: and though I rather advised him to submit to the operation, yet there were traits in his constitution which induced me not to press it, and made me pleased that he was previously bent against it.

He had a highly sallow, diseased complection, a general want of muscular flesh and firmness; a frequent colic, sometimes attended with a diarrhœa, and oftentimes with an obstinate constipation.

During the space of twelve months he took a variety of medicines, and saw a number of practitioners, but found no relief, neither did the testicle in that time suffer any material alteration, or the process become in the least changed. He died of a stubborn and painful dysentery; and when he was opened, his mesentery was found full of large, hard, schirrous knots; all the lymphatic glands about the receptaculum chyli remarkably diseased, and the liver greatly enlarged and indurated.

